# Usability, sense of presence, and performance of a virtual reality emotion recognition task

**DOI:** 10.1371/journal.pone.0330084

**Published:** 2025-08-12

**Authors:** David Pérez-Ferrara, Yvonne Flores-Medina, Michelle Gómez, Guillermina Yáñez-Téllez, Alejandra Mondragón-Maya

**Affiliations:** 1 Facultad de Estudios Superiores Iztacala, Universidad Nacional Autónoma de México, Tlalnepantla de Baz, Estado de México, México; 2 Instituto Nacional de Psiquiatría “Ramón de la Fuente Muñiz”, Ciudad de México, México; Gachon University, KOREA, REPUBLIC OF

## Abstract

Virtual reality (VR) has been proposed as a tool that could fulfill some of the limitations in emotion recognition (ER) assessment. Our research group created a VR task (*VR-Tóol*) for ER assessment. The aims of this study were to assess the usability, sense of presence, and acceptability of the *VR-Tóol*; to describe the performance of a *VR-Tóol* setup task and to examine the association between *VR-Tóol* performance and empathy. Forty-six healthy participants were assessed. A semi-structured interview, the Symptom Checklist-90 (SCL-90), the Cognitive and Affective Empathy Test (TECA), and the ICT Use Questionnaire (ICTUQ) were administered. Subsequently, a *VR-Tóol* protocol was applied. Finally, the System Usability Scale (SUS)*,* Igroup Presence Questionnaire (IPQ), and a Motivation Questionnaire were administered. The SUS total score showed acceptable parameters; the IPQ mean scores reached marginally acceptable scores in all subscales, excepting for the Involvement subscale, which achieved non-acceptable parameters. The SSQ indicated the presence of negligible symptoms related to the VR exposure. Regarding performance, participants achieved 83.3% hits. A substantial accuracy rate was met in contempt, admiration, happiness, surprise, and compassion. Significant positive correlations were observed between *VR-Tóol* hits and TECA’s Perspective taking (PT) score (Spearman’s rho = 0.500, p < .001) and Total score (Spearman’s rho = 0.398, p = .007). In conclusion, *VR-Tóol* exhibits favorable usability and sense of presence properties, negligible cybersickness symptoms, and an overall positive experience. We found evidence of the relationship between ER and empathy. This work elucidates the potential utility of *VR-Tóol* for ER assessment.

## Introduction

Emotion recognition (ER) has been defined as the ability to identify emotional states in others through social cues like facial expressions, body postures, and prosody [1]. ER is crucial for its socio-evolutionary role in facilitating adaptive social interactions [2]. On this basis, ER is considered a basic process which allows other more complex social abilities to develop, like emotion regulation, empathy, or Theory of Mind (ToM) [3]. Regarding the emotional processing domain, some models have suggested that ER is the first required component in a chain of subsequent processes which enable the individual to regulate and manage emotions in oneself and in respect to others [4,5]. Moreover, some models on empathy propose that ER occurs when the perceiver must tag the emotional state of the other, to display an empathetic response [6]. Finally, evidence has shown that ER brings essential information for the inference of causal attributions related to the identified emotional state of the others, thus contributing to ToM [3].

Research on ER has provided a wide variety of assessment methods according to different theoretical frameworks. According to Paiva-Silva et al. [7], the most common assessment approach consists of the presentation of static photographs containing facial expressions of the six basic emotions, as proposed by Ekman [8]: happiness, anger, disgust, fear, sadness, and surprise. Although this approach has provided valuable information in clinical and research contexts, some limitations have been identified regarding its ecological validity. Daily life ER is accomplished with dynamic stimuli, which provide changing facial gesticulations to express an emotion. Such changes may vary in intensity and motion patterns, which cannot be captured in the static stimuli. Moreover, as mentioned above, this approach includes only the identification of six emotions. However, the emotional phenomenon is not restricted to basic emotions, since a wider set of emotions, denominated secondary or complex, constitutes a significant portion of it [2,9]. Taken together, the evidence points out that the traditional and most used approach for ER assessment needs to be updated and upgraded to provide a wider perspective of the ER construct. Considering this, the utilization of innovative technologies that could facilitate the use of dynamic and more ecological stimuli on ER research could significantly enrich the current state-of-art on the topic. Among the available emerging technologies, virtual reality (VR) stands out as a suitable and feasible option since dynamic stimuli within a controlled real-life-like environment can be employed. Such an environment may enhance ecological validity while preventing the exposure of the participants to real unsafe or dangerous scenarios that may compromise their well-being. Moreover, in ER research, gesticulation and expression intensity can be included and manipulated for experimental purposes, maintaining strong control of the involved variables. In this regard, VR brings the flexibility to program and run different paradigms according to diverse purposes or even to the participants’ needs or specific capabilities. The utilization of VR also reduces the cognitive demand on the evaluator, since stimuli can be automatically presented according to established parameters, and automated performance and response times registration can be obtained, preventing human errors or biases [10].

In light of the traditional ER assessment methods limitations and the potential benefits of VR to address some of them, our research group created a novel VR software for ER assessment denominated *VR-Tóol*. This innovative software includes dynamic emotional expressions that can be presented in three intensity levels (i.e., mild, moderate, and high). The software includes six basic emotions, plus six complex emotions (i.e., admiration, arrogance, compassion, pain, contempt, and shame), which are barely studied in ER research [7]. It also includes four response options (i.e., forced multiple choice, forced multiple choice with feedback, dimensions of core affect, and free verbal response) which can be arrayed according to the objectives and approach of the assessment. Finally, performance and response times can be automatically recorded. All these features allow to measure ER in a more precise and ecological fashion, expanding the assessment to a wider array of emotions, and enabling the evaluation of ER in different response manners according to the needs and approaches of the study. In the present manuscript, we introduce *VR-Tóol,* and provide the first exploratory data about its usability, sense of presence, acceptability, and performance in a sample of healthy adults.

### Literature review

#### ER approaches in psychological research.

Different approaches for the study of ER have emerged: the basic emotions theory [8], the dimensional theory [11], and the theory of constructed emotion [12]. Firstly, the basic emotions theory proposes that there are six basic emotions linked to adaptive biological functions and are assumed to be culturally universal, these basic emotions are represented by a distinct neural and psychophysiological pattern [8]. Secondly, the dimensional theory postulates that emotions are based on a continuum of fundamental biological dimensions; valence (positive/negative) and arousal (calm/tension). The combination of both elements will facilitate a behavioral response that will lie between the approach or avoidance of emotional stimuli. Different combinations of these dimensions create emotions that do not fit specific categories [11,13]. Lastly, the constructed emotion theory argues that emotional experiences and perceptions are constructed from basic operations, such as body sensations and feelings (core affect) and context-derived sensations (exteroceptive information) that acquire meaning from previous experiences (conceptual knowledge), including the emotional categories encoded through language [12]. Xu, Peng, Luo, and Gong [14] conducted a meta-analysis to explore the brain regions and networks associated with ER through functional neuroimaging techniques. The purpose of the study was to test the neurobiological underpinnings of basic, dimensional, and constructed emotion theoretical models. The meta-analysis comprised 3138 participants from 141 studies. The researchers observed common connectivity patterns across the different emotional categories and dimensions, whereas the amygdala was the hub of a distributed network for the representation of core affect. These findings are inconsistent with the assumptions of the basic emotion theory, which posits that each emotion has a unique neural representation. Interestingly, it also contradicts the dimensional assumption that bipolar neural networks would engage in opposition according to the valence and arousal of the emotion, since no differential engagement was observed across networks at opposite dimensions (e.g., positive versus negative valence). Finally, the authors concluded that the observed neural patterns across the different studies supported the constructed emotion model, in which general-domain brain networks rather than specific ones, are engaged and their combination provides further behavioral specificity that enables ER. This neurobiological evidence points out that ER processing is more complex than both basic and dimensional approaches, highlighting the need to update its assessment, in which the inclusion of diverse stimuli, variables, and assessment methods could provide a wider perspective for its understanding.

#### Assessment methods for ER.

A variety of paradigms and tasks have been developed for the assessment of ER. There is considerable diversity among these instruments regarding the type of stimuli presented (S1 Table) and the type of response required (S2 Table).

Nevertheless, important limitations have been detected among these assessment methods. As mentioned before, most studies have employed a basic emotions approach, which focuses only on six emotions (i.e., anger, disgust, fear, happiness, sadness, and surprise), despite the evidence of wider emotional arrangements that contribute to adaptative responses and include specific facial expressions as well [9,13]. Although the existence of complex or secondary emotions is evident, the fact that they are not considered universally recognizable, but more related to culture and experience, has prevented their study [15,16]. Additionally, previous research has employed the presentation of facial expressions in isolation from social context, which is an essential inherent feature of the processing of facial expressions [9,13,17]. As reviewed earlier, emotions are strongly linked to social interactions, so environmental contextualization should be included in ER stimuli, particularly when measuring complex emotions [9,15]. Furthermore, ER assessment has focused almost exclusively on facial expressions, regardless of the existence of body gestures that tend to co-occur the facial expression, thus reinforcing the emotional reaction (e.g., hands and head movements, chest expansion, etc.) [15]. It is notable that most studies do not include dynamic stimuli, despite the evidence indicating that the neural responses are stronger to dynamic, complex, and naturalistic stimuli than to simple and static ones [13]. In consideration of the aforementioned, VR has been proposed as a potential tool for addressing some of the limitations found in the traditional ER assessment, as VR has the capacity to create dynamic, contextualized, and controlled environments. Thereby, offering a promising avenue for enhancing the ER assessment.

#### VR as an assessment tool.

VR has been defined as a type of advanced interaction between a person and a computer, which enables a more naturalistic interplay within the virtual environment and its characters. It involves real-time stimulation and interaction of an individual situated within an artificial environment, facilitated through the engagement of multiple sensory channels [18]. The use of VR enables the creation of environments that closely resemble real-world scenarios and their associated demands [18], which cannot be replicated in paper-and-pencil tests [9]. Moreover, VR can facilitate the preservation of robust experimental control and accommodate diverse experimental conditions or paradigms [18]. Furthermore, automated behavioral data can be obtained when running a VR task, thereby reducing the probability of human errors or heterogeneity among evaluators [10]. As mentioned by Abbas Shah et al. [19], VR is a valuable tool that enables the creation of engaging and interactive experiences that have the potential to enhance a better understanding of the experience.

With respect to the use of immersive VR for ER assessment, several studies have been published in this field. Garaets et al. [20] compared the performance of healthy subjects in VR and traditional ER tasks. The researchers employed dynamic stimuli comprising six basic emotions presented in different avatars. The authors concluded that the patterns of recognition and the proportion of correct responses were highly similar between the photo, video, and VR tasks. Moreover, Frazen et al. [21] employed this set of facial expressions with avatars to evaluate a sample of bullying victims, wherein they identified misattribution of anger in neutral faces. In a further study [22], the authors employed dynamic stimuli comprising the six basic emotions and observed a correct response rate of 90.3%. The facial expressions were previously validated by Fernándes-Sotos et al. [23] using non-immersive VR. In a recent study, Vicente-Querol et al. [24] concluded that the level of immersion and resolution of the device are positively associated with ER accuracy. In this regard, Zhang et al. [25] found that avatars presented in a 3D format showed increased brain activation, particularly in frontal, temporal, and occipital regions. Recently, González-Gualda et al. [26] introduced a VR task for ER assessment and analyzed eye gaze patterns in a healthy sample. The authors identified different eye gaze patterns according to age and gender groups when recognizing the six basic emotions. Furthermore, ER assessment with VR has also been proposed to be used in psychiatric samples like schizophrenia [27], providing promising and insightful results.

At present, VR is being employed for training purposes for ER and other social cognition processes. A scoping review of social cognition interventions, including ER, in patients with schizophrenia reported promising results. However, the authors noted considerable heterogeneity in the characteristics of the interventions and the methodologies used in the studies [28]. Conversely, Farashi et al. [29] conducted a meta-analysis, which identified a positive effect of computerized training and VR on ER in patients with autism spectrum disorder.

Notwithstanding the significant contributions of existing studies to this field, several limitations of VR tasks for ER research remain unaddressed. For instance, numerous studies that have created virtual stimuli for ER have concentrated only on the six basic emotions, without accounting for the body posture, the intensity of the emotional expressions, and have relied on forced multiple-choice responses [20,21,23,30,31].

#### Usability and sense of presence in VR tools: VR-CHECK.

Despite the growing use of VR technology in fields such as psychology, neuropsychology, and neuroscience, there is currently no consensus regarding the key factors to consider when developing a VR cognitive assessment or rehabilitation tasks. In this context, Krohn et al. [10] developed a framework for multidimensional assessment of VR cognitive paradigms, designated as VR-CHECK. The framework proposes that a total of 10 relevant dimensions should be considered when developing and evaluating VR cognitive tasks: 1) Domain specificity; 2) Ecological relevance; 3) Technical feasibility; 4) User feasibility; 5) User motivation; 6) Task adaptability; 7) Performance quantification; 8) Immersive capacities; 9) Training feasibility; and 10) Predictable pitfalls.

Considering the VR-CHECK framework, it is relevant to assess some variables that are fundamental to develop VR cognitive tasks. In this regard, the technical and user feasibility is associated with the software usability. Usability is defined as the level of comprehension, learning potential, utility, and attractiveness of the software as perceived and measured by the user. Usability assessment provides insight into the participants’ perceptions of the software’s ease of use and interaction, as well as their comprehension of the instructions and the task [32]. Krohn et al. [10] provide a definition of immersive capacities as technical properties that support natural sensorimotor contingencies within a VR system. Immersive capacities evaluate the degree of immersion as specified by task factors and the VR system necessary to run the task. The concepts of immersion capabilities and sense of presence are distinct yet related. Sense of presence has been defined as the subjective sensation of being present in a virtual environment [33]. Schubert, Friedmann, and Regenbrecht [34] identified three key factors that contribute to the sense of presence experienced by users: involvement, spatial presence, and realism. It is pertinent to evaluate the sense of presence and immersive capacities, given the advent of cutting-edge VR technology. This has led to the hypothesis that an enhanced degree of presence could prove advantageous in a multitude of domains, including diagnostic assessment, cognitive training outcomes, and user experience [35,36]. There is emerging evidence suggesting that heightened presence may positively influence participants’ cognitive performance [37]. Nevertheless, the potential advantages of augmented presence in clinical assessment and treatment remain to be investigated.

In the present manuscript, we introduce a novel VR software for ER assessment, denominated *VR-Tóol*. This software facilitates the assessment of the six basic emotions and six complex emotions, with various degrees of intensity (i.e., mild, moderate, high), and some of the emotions also integrate corporal movements. It is displayed in an immersive VR context and offers a high degree of flexibility by supporting various response options, including multiple-choice, multiple-choice with feedback, dimensional, and free verbal response. Additionally, it enables the creation of multiple automated protocols and real-time experimentation. In the present study, we sought to explore some features of *VR-Tóol*. The research questions leading this pilot study are:

1. Which usability, sense of presence, and acceptability parameters will be obtained from a *VR-Tóol* setup task applied to a sample of healthy adults?
2. Which performance rates, in terms of hits, misses, and response times will be obtained with a *VR-Tóol* setup task applied to a sample of healthy adults?
3. What is the association between *VR-Tóol* performance and scores from an empathy questionnaire in a sample of healthy adults?

The objective of this pilot study is to provide evidence on the usability, sense of presence, and acceptability (in terms of cybersickness symptomatology and satisfaction) of the *VR-Tóol.* Further, we aimed to explore and describe the performance of a *VR-Tóol* setup task in terms of hits, misses, and response times, and to explore the association between *VR-Tóol* performance and scores of an empathy questionnaire. We expected to obtain acceptable usability and sense of presence parameters from the *VR-Tóol* setup task. Furthermore, we expected to observe positive *VR-Tóol* acceptability parameters, in terms of non-significant or negligible cybersickness symptoms, and adequate satisfaction reports from the users. Regarding *VR-Tóol* performance from the setup task, we predicted that similar performance rates to those reported in traditional tasks would be obtained. Finally, given the theoretical relationship between ER and empathy, we anticipated to observe significant positive associations between *VR-Tóol* performance and empathy scores.

## Materials and methods

This study employs a non-experimental, mixed cross-sectional methodology.

### Participants

A total of 51 participants were recruited from May 2^nd^ to May 26^th^, 2023, at the Facultad de Estudios Superiores Iztacala (FESI). A non-randomized convenience sampling was used. To be included in the study, participants were required to be at least 18 years of age, male or female, and to have completed a minimum of six years of formal education. The decision to participate in the study was entirely voluntary. Prior to data collection, all participants were required to sign a written informed consent form. The exclusion criteria comprised the presence of any neurological or psychiatric condition, a score equal to or greater than 70 on the SCL-90’s General Severity Score (GSI), or the presence of any self-reported perceptual disability that could impede the assessment process.

**Fig 1:**
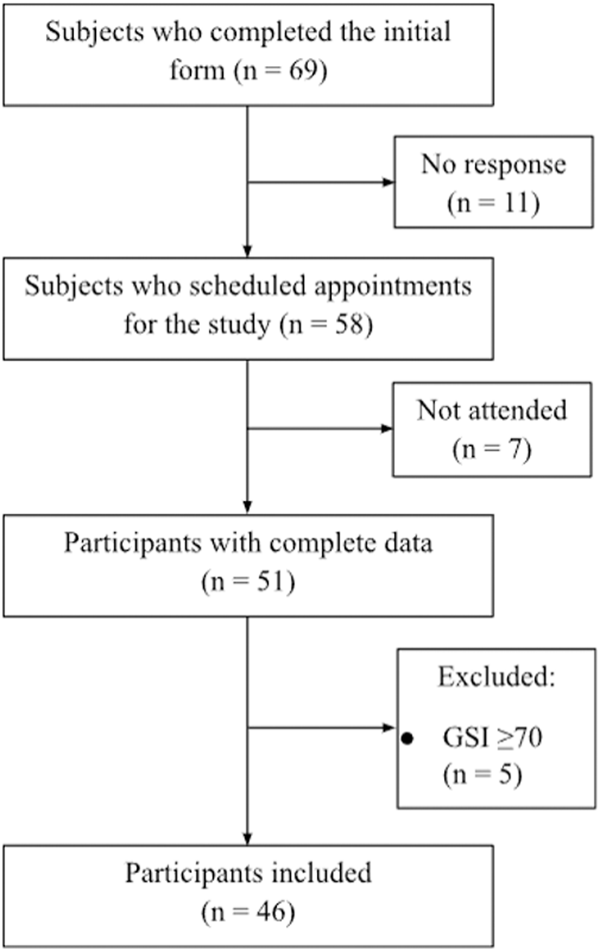
Flow diagram of the recruitment process.

Five participants were excluded from the analysis due to obtaining total scores equal to or greater than 70 on the GSI scale of the SCL-90. As a result, the final sample comprised 46 subjects. Participants were eliminated from the study if they experienced significant cybersickness symptoms associated with the VR task. No participants reported experiencing moderate to severe symptoms of cybersickness during the assessment; therefore, all of them completed the protocol. The present study adheres to the requirements of the Helsinki Declaration and was approved by the Ethics Committee of FESI (Register number: CE/FES/2021/1384). Fig 1 illustrates the sample’s recruitment process.

### Materials and instruments

#### ER assessment: *VR-Tóol.*

**Fig 2:**
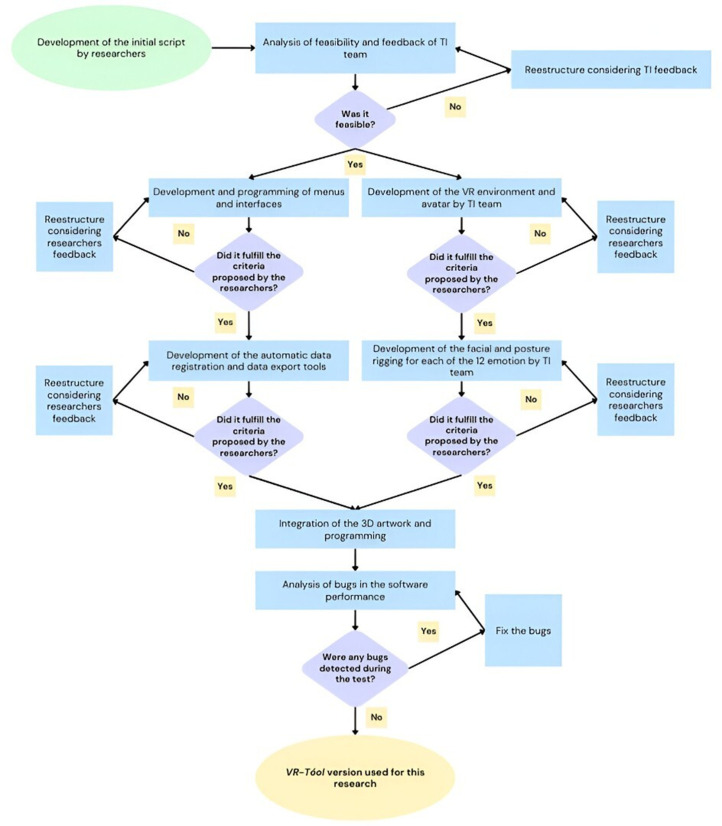
Flow diagram illustrating the developmental process of the *VR-Tóol.*

***Description of the software:*** *VR-Tóol* (*óol* is a derivative term from Mayan that is associated with mood, volition, movement, which regulates mood states, thoughts, and behavior) [38] is a system for assessing basic and complex ER with immersive VR. The system was developed in Unity. The system enables the assessment of the ER of six basic emotions (i.e., happiness, sadness, disgust, anger, fear, and surprise) and six complex emotions (i.e., admiration, arrogance, compassion, pain, contempt, and shame) in a virtual avatar. Additionally, three different intensities are available for selection (i.e., mild, moderate, and high). Intensity was determined by making the prototypical gestures associated with each emotion less or more pronounced. The emotional expressions were developed in accordance to the Facial Action Coding System (FACS) [9] and the Facial and Body Emotion Recognition Test (REFyC) [39]. It should be noted that complex emotions are accompanied by body movements, whereas basic emotions are manifested solely in facial expressions. This program assesses ER through four response options, namely forced multiple choice, forced multiple choice with feedback, dimensions of core affect, and free verbal response. Additionally, it provides the flexibility to develop protocols or generate stimuli in real time, tailored to the specific needs of the researcher or therapist. Furthermore, it allows for the automatic recording of responses and response times for each stimulus. Fig 2 illustrates the developing process of *VR-Tóol* software.

**Fig 3:**
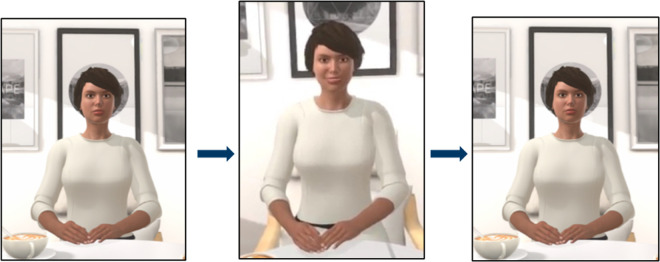
Sequence of the *VR-Tóol* task.

**Fig 4:**
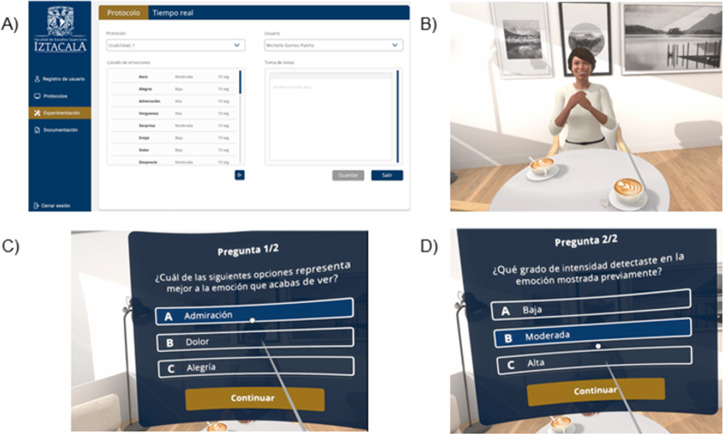
Screenshots from *VR-Tóol.* A) Platform screenshot, this figure shows the platform used by the evaluator during the application of the protocols; B) Participant view screenshot during the expression of an emotion; C) Response options screenshot for identifying the emotion; D) Response options for identifying the intensity.

***Description of the task:*** In the task, participants are placed in front of the female avatar in a VR living room. Before the task begins, the avatar will assume a neutral posture. Once the task has begun, the participant will hear a beep, which will prompt the avatar to generate an emotional expression. Subsequently, the avatar will return to a neutral position (Fig 3). After the avatar’s facial and body gestures are presented, participants must identify the emotional expression (Fig 4). Once the participant has responded, a new stimulus will be presented in accordance to the sequence.

***Description of the protocol:*** For the current study, a 36-item protocol was utilized. All 12 emotions and each of their intensities (mild, moderate, or high) were presented in a pseudo-random pre-established order. Participants were required to provide their responses within a multiple-choice format, which included the target word and two randomly selected options, once the avatar’s emotional expression concluded. First, they had to identify the emotion within an arrangement of three choices. Once they responded, a second arrangement of three options regarding the expressed emotion intensity (i.e., mild, moderate, high) had to be responded. Correct and incorrect responses, as well as response times, were recorded using the automated software (Fig 4).

A desktop computer with a Ryzen 7 5700G processor, NVIDIA RTX 3070 GPU, and 32 GB of RAM was utilized to run the program, while an Oculus Quest 2 headset was employed for immersion.

#### Clinical assessment.

***Semi-structured Interview:*** A semi-structured interview format was developed to collect sociodemographic data and verify inclusion/exclusion criteria (S3 File).

***Symptom Checklist SCL-90-R* [40]:** This screening tool quantifies psychopathological symptoms that may indicate the presence of psychological distress or a psychiatric disorder. The instrument was used to verify the presence of any potential psychiatric condition in the sample. It provides information clustered in ten psychiatric categories: Somatization (SOM), Obsession-compulsion (OC), Interpersonal sensitivity (SEN), Depression (DEP), Anxiety (ANX), Hostility (HOS), Phobic anxiety (PHO), Paranoid ideation (PAR), Psychoticism (PSY), and Additional symptoms (ADD). A general score (GSI) can be obtained as well, which was used for inclusion/exclusion criteria. Cruz et al. [41] presented evidence indicating that the GSI (Cronbach’s alpha = 0.96) and nine of the eleven subscales (Cronbach’s alpha = > 0.7–0.85) of the SCL-90 display satisfactory reliability parameters when assessed among individuals of Mexican heritage.

***Cognitive and Affective Empathy Test (TECA)* [42]:** This instrument measures a global dimension of empathy and provides four empathy-related subscales. This scale has a good reliability for the total score (Cronbach’s alpha = 0.86), and for the subscales of Perspective Taking (PT; Cronbach’s alpha = 0.70), Emotional Understanding (EU; Cronbach’s alpha = 0.74), Empathic Stress (ES; Cronbach’s alpha = 0.78) and Empathic Joy (EJ; Cronbach’s alpha = 0.75).

#### Usability, presence, and acceptability assessment.

***System Usability Scale (SUS)* [**32,43**]:** A 5-point Likert scale comprising 10 items used to measure participants’ subjective perception of program usability. Higher scores indicate better usability parameters. Bangor et al. [44] found that the SUS was highly reliable (Cronbach’s alpha = 0.91) and useful over a wide range of interface types. In a separate study [45], the authors report the following cut-off points: ≤ 50 = Not acceptable; 50–70 = Marginally Acceptable; ≥ 71 = Acceptable. These parameters were utilized for the interpretation of the resulting data.

***Igroup Presence Questionnaire (IPQ)* [46]:** This questionnaire measures the sense of presence experienced in virtual environments. It consists of 14 items rated on a 7-point Likert scale. A total Presence score and three factors can be obtained. Higher scores indicate better presence parameters. The authors report good reliability [46] and cut-off points [47] for the total scale (Cronbach’s alpha = 0.85; Presence score ≤ 3.46 = Not acceptable; ≥ 3.47 = Marginally acceptable; ≥ 3.86 = Acceptable), and for the subscales of Spatial Presence (Cronbach’s alpha = 0.80; Spatial presence score ≤ 4 = Not acceptable; ≥ 4.01 = Marginally acceptable; ≥ 4.50 = Acceptable), Involvement (Cronbach’s alpha = 0.76; Involvement score ≤ 3.37 = Not acceptable; ≥ 3.38 = Marginally acceptable; ≥ 4 = Acceptable) and Realism (Cronbach’s alpha = 0.68; Realism score ≤ 2.62 = Not acceptable; ≥ 2.63 = Marginally acceptable; ≥ 3.38 = Acceptable). These parameters were utilized for the interpretation of the resulting data.

***Simulator Sickness Questionnaire (SSQ)* [**48,49**]:** This questionnaire assesses 16 symptoms related to cybersickness on a 4-point Likert scale. The symptoms are clustered into three dimensions. Higher scores indicate more severe symptoms. Sevinc and Berkman [50] reported a good reliability for the total scale (Cronbach’s alpha = 0.94), and for the subscales of nausea (Cronbach’s alpha = 0.84), oculomotor (Cronbach’s alpha = 0.91), and disorientation (Cronbach’s alpha = 0.88). Brown et al. [51] reported the following cut-off points: 0 = No symptoms; ≤ 5 = Negligible symptoms; 5–10 = Minimal symptoms; 10–15 = Significant symptoms; 15–20 = Symptoms are a concern; ≥ 20 = A bad intervention. These parameters were utilized for the interpretation of the resulting data.

***ICT Use Questionnaire* (ICTUQ) [52]:** This questionnaire measures the type and frequency of technology use on a 4-point Likert scale. It includes 17 items divided into three subscales. Higher scores are associated with a higher frequency of ICT use. Coppari et al. [52] reported a good reliability for the total scale (Cronbach’s alpha = 0.79), and for the subscales of Virtual information/education (Cronbach’s alpha = 0.69), Mediated communication/interaction (Cronbach’s alpha = 0.91), and Entertainment/leisure (Cronbach’s alpha = 0.57).

***Motivation and Satisfaction Questionnaire* (MSQ):** This instrument was designed by our research group to delve into participants’ opinions about their experience using *VR-Tóol*. It includes eleven multiple-choice items, of which items 6, 9, and 11 are inverted, and four open-ended questions. Scores on the motivation and satisfaction questionnaire ranged from zero to six. For items one to three, zero indicated “not at all” and six indicated “very much.” For the other items, zero indicated “strongly disagree” and six indicated “strongly agree” (S4 File).

### Procedure

Social media and printed advertisements were posted within the FESI for sample recruitment. In-person appointments were scheduled based on the availability of potential participants. Once at the session, the aims of the study were explained, and participants signed the informed consent letter. The semi-structured interview, SCL-90-R, TECA, and the ICTUQ were administered in that order. Subsequently, the Oculus headset was placed on participants, allowing a few minutes for exploring the VR environment and ensuring proper fit of the headset. Task instructions were provided verbally, and three practice items were included. Thereafter, the experimental protocol was applied and lasted approximately 15 minutes. Throughout this period, participants remained seated in a rotating chair within a cubicle at FESI. After completing the VR task, participants were asked to complete the remaining assessment scales: SUS, IPQ, SSQ, and MSQ. The entire session lasted approximately one hour.

Descriptive statistics were used to analyze demographic data, usability, sense of presence, acceptability, and *VR-Tóol* performance. Furthermore, a qualitative word frequency analysis was conducted to explore responses to the open-ended questions of the MSQ. Lastly, partial Spearman correlations were calculated to examine the relationship between ER and empathy. Since psychiatric symptomatology related to psychotic disorders has been reported as significantly detrimental to ER [53], we adjusted the analysis by the PSY domain of the SCL-90-R. For quantitative analyses, JASP version 0.17.1.0 was utilized, and word clouds were generated using MATLAB R2021b.

## Results

### Descriptive statistics

The sample consisted of 46 participants with a mean age of 30.48 (SD = 11.12) and an average of 15.9 (SD = 3.3) years of schooling. Sixty-three percent of the sample identified themselves as female, and the majority were engaged in formal employment (39%), undergraduate students (31%), or graduate students (22%). Notably, 59% of the participants had never used immersive VR in any context.

### Usability, sense of presence, and cybersickness

The results and qualitative interpretations of the SUS, IPQ, and SSQ are presented in detail in Table 1. Notably, SUS mean score reached acceptable parameters, IPQ scores reached marginally acceptable parameters in all subscales, excepting for the Involvement subscale, whereas a non-acceptable score was met. Finally, SSQ mean score corresponded to negligible symptoms. A visual representation of the mean of the SSQ scales considering the cut-off points proposed by Brown et al. [49], can be found in Supplementary Material 5 (S5 File).

**Table 1:** Descriptive statistics of the SUS, IPQ and SSQ.

	n	Mean	SD	Interpretation
SUS Total	46	85.87	9.67	*Acceptable* ^*a*^
IPQ – SP	46	4.29	1.764	*Marginally Acceptable* ^*b*^
IPQ – INV	46	3.17	1.89	*Not acceptable* ^*b*^
IPQ – REA	46	2.88	1.837	*Marginally Acceptable* ^*b*^
IPQ – P	46	3.71	1.915	*Marginally Acceptable* ^*b*^
SSQ Total	46	4.848	5.287	*Negligible symptoms* ^*c*^
Nausea	46	1.13	1.31	
Oculomotor	46	3.283	3.716	
Disorientation	46	2.304	3.01	

SSQ = Simulator Sickness Questionnaire; SUS = System Usability Scale; IPQ = Igroup Presence Questionnaire; SP = Spatial Presence; INV = Involvement; REA = Realism; P = Presence.

a = Bangor et al. [44].

b = Melo et al. [46].

c = Brown et al. [49].

### Motivation and satisfaction

**Fig 5:**
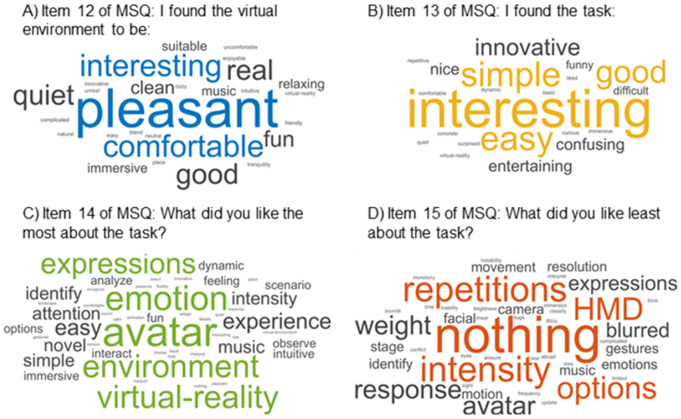
Word clouds of the MSQ open questions. A) Item 12 of MSQ: I found the virtual environment to be; B) Item 13 of MSQ: I found the task; C) Item 14 of MSQ: What did you like the most about the task?; D) Item 15 of MSQ: What did you like least about the task.

Participants reported liking the virtual environment (MSQ-1, Mean = 5.022, SD = 0.856) and enjoying participating in the task (MSQ-2, Mean = 5.391, SD = 1.105). They expressed a willingness to participate in the task again (MSQ-3, Mean = 5.326, SD = 1.117) and indicated that they would recommend their family members to participate as well (MSQ-10, Mean = 5.609, SD = 0.881) (Table 2). Additionally, participants found the virtual environment pleasant, comfortable, and interesting. Regarding the task, they found it interesting and simple (Fig 5).

**Table 2:** Descriptive statistics of the MSQ.

Item	n	Mean	SD
How much did you like the virtual environment?	46	5.022	1.105
How much did you enjoy participating in the VR task?	46	5.391	0.856
How much would you like to participate in this VR task again?	46	5.326	1.117
The avatar’s facial expressions looked real to me.	46	3.891	1.233
I am willing to participate in this task again in the future.	46	5.717	0.688
The emotional expressions of the avatar seemed fake to me.	46	2.087	1.603
I found the response options to be appropriate	46	4.37	1.451
The avatar’s emotional expressions seemed natural to me.	46	3.63	1.356
I experienced discomfort after performing the task.	46	1.022	1.844
I would recommend my family and friends to participate in this task.	46	5.609	0.881
I was confused by the format of the response options.	46	1.348	1.864

MSQ = Motivation and Satisfaction Questionnaire. The MSQ employs a 7-point Likert scale, with scores ranging from 0 to 6.

### *VR-Tóol* performance

The mean total score on the *VR-Tóol* was 30 (SD = 3.32). Participants achieved 83.3% correct responses. The average response time was 10.2 s (SD = 2.39 s). When grouping the items according to intensity, high intensity was the easiest to identify (90%), followed by moderate intensity (86.1%), and finally low intensity (73.9%). The emotions with the highest percentage of correct responses were contempt (92%), admiration (91.3%), happiness (88.4%), surprise (87%), and compassion (87%) (Table 3). Finally, the identification of intensity resulted in only 52.6% correct responses and a mean total score of 18.93. For a more in-depth review of participants’ performance by emotion and intensity, please refer to Supplementary Material 6 (S6 File).

**Table 3:** Description of *VR-Tóol* results by emotion and intensity.

Emotion	Intensity	Pt	% Correct answers	Mean Time (s)
Total correct score		1377	83.3	10.2
	Mild	408	73.9	10.69
	Moderate	435	86.1	10.09
	High	497	90	9.83
Happiness		122	88.4	9.31
	Mild	38	82.6	10.05
	Moderate	41	89.1	9.18
	High	43	93.5	8.71
Anger		103	74.6	9.31
	Mild	23	50	11.68
	Moderate	39	84.8	9.55
	High	41	89.1	9.03
Surprise		120	87	9.89
	Mild	36	78.3	10.20
	Moderate	39	84.8	10.55
	High	45	97.8	8.9
Sadness		112	81.2	10.005
	Mild	33	71.7	9.57
	Moderate	39	84.8	9.72
	High	40	86.9	10.71
Disgust		117	84.8	9.54
	Mild	30	65.2	10.27
	Moderate	41	89.1	9.99
	High	46	100	8.38
Fear		115	83.3	10.19
	Mild	32	69.6	10.18
	Moderate	42	91.3	10.18
	High	41	89.1	10.20
Admiration		123	91.3	9.51
	Mild	44	95.6	9.38
	Moderate	43	93.5	9.18
	High	39	84.8	9.96
Arrogance		96	69.6	11.17
	Mild	31	67.4	11.34
	Moderate	30	65.2	11.66
	High	35	76.1	10.47
Compassion		120	87	10.25
	Mild	37	80.4	11.64
	Moderate	39	84.8	9.57
	High	44	95.7	9.53
Pain		105	76.1	11.51
	Mild	27	58.7	11.59
	Moderate	37	80.4	10.07
	High	41	89.1	10.62
Shame		117	84.8	11.61
	Mild	38	82.6	11.77
	Moderate	40	87	10.99
	High	39	84.8	12.07
Contempt		127	92	10.16
	Mild	39	84.8	10.72
	Moderate	45	97.8	10.42
	High	43	93.5	9.33

The percentages were calculated considering the sum of points for the entire sample. For the total score, a total of 1656 (36 items x 46 participants) was considered, for the total intensities 552 (12 items x 46 participants) were considered for each one (mild, moderate, and high). Regarding the percentages by emotions, a total of 138 (3 items x 46 participants) and for the intensities 46 (1 item x 46 participants) were considered for each one (mild, moderate, and high).

### *VR-Tóol* and empathy

Finally, significant positive correlations were observed between the total number of correct answers on the *VR-Tóol* and the PT score (r = 0.500; p < .001) as well as the total score (r = 0.398; p = 0.007) on the TECA. Additionally, a positive correlation was found between the average time spent on the *VR-Tóol* and age (r = 0.332; p = 0.031) (Table 4). The correlation plots of the significant correlations can be found in Supplemental Material 7 (S7 File).

**Table 4:** Spearman correlations between *VR-Tóol,* TECA, Schooling, and ICTUQ.

Variable		TECA Total	TECA PT	TECA EC	TECA ES	TECA EJ	Age	Years of education	ICTUQ Total
*VR-Tóol* Time	Spearman’s rho	−0.254	−0.232	−0.243	0.034	−0.114	0.332*	−0.234	−0.292
	p-value	0.092	0.125	0.107	0.823	0.456	0.031	0.122	0.051
*VR-Tóol* Intensity	Spearman’s rho	0.107	0.302*	0	−0.129	0.059	−0.092	0.121	0.132
	p-value	0.482	0.044	0.996	0.398	0.698	0.55	0.429	0.388
*VR-Tóol* Correct	Spearman’s rho	0.398**	0.5***	0.214	0.251	0.117	−0.184	0.228	0.152
	p-value	0.007	<.001	0.157	0.096	0.245	0.226	0.131	0.32

TECA = Cognitive and Affective Empathy Test; PT = Perspective taking; EC = Emotional understanding; ES = Empathic Stress; EJ = Empathic Joy.

All tests one-tailed, for positive correlation.

Conditioned on variables: SCL_90_PSY.

* p < .05, ** p < .01, *** p < .001, one-tailed.

## Discussion

### Usability, sense of presence, and acceptability

The objectives of the present study were fully met. The first aim was to explore *VR-Tóol*’s usability, sense of presence, and acceptability in a sample of healthy adults. The obtained usability score for *VR-Tóol* reached acceptable parameters according to Bangor et al. [45]. As previously stated, usability is defined as the extent to which a software is perceived as easy to use, friendly, comprehensible, and attractive by real users. Thus, the scores obtained on this matter indicate that the task was easy to understand and to perform, so the participants reported a general positive VR experience using *VR-Tóol*. Regarding sense of presence, we followed Melo et al. [47] interpretation guidelines. Most scores reached “marginally acceptable” parameters, but the Involvement subscale, which showed a “not acceptable” score. As mentioned before, sense of presence involves the subjective experience of being present into a VR environment. The “marginally acceptable” scores reached in most subscales were expectable, since the visual art of the VR environment was not intended to be hyper realistic, and the characteristics and aims of the task provided limited spatial manipulation for the user’s experience. However, the Involvement subscale, which assessed the degree of engagement into the VR environment, showed “not acceptable” scores, indicating that the users were not entirely embedded into the VR environment. There may be some factors that could prevent the user’s involvement into the VR environment (e.g., lack of interaction between the user and the avatar, no inclusion of verbal cues or dialogues, etc.). Interactivity has been identified as being essential in enhancing presence. The provision of interaction-rich environments offers users opportunities for exploration and active participation, thereby fostering enhanced immersion [54]. Such variables should be considered for future *VR-Tóol* versions to enhance the user’s sense of presence. Acceptability was measured through a cybersickness symptomatology scale and a questionnaire that explored the participants’ experience with *VR-Tóol* to complement the collected information from the other instruments. According to Brown et al. [51], the experienced symptoms related to the *VR-Tóol* setup task used in the present study, were considered as “negligible symptoms”, meaning their frequency and severity are minimal and clinically non-significant. Although evidence has indicated that the use of headsets for immersive VR is related to higher cybersickness [55], other authors have suggested that the content of the VR environment seems to be a determinant factor related to cybersickness symptoms [56]. On this regard, Oh and Son [57] observed that variables included in a VR environment, like rotating cameras, acceleration, and long scene duration, are some of the VR content features that are more related to higher levels of cybersickness. In contrast, minimalist scenes, with little movement and stable scenarios, seem to be related to lower levels of cybersickness [56]. In the present study, the environment of *VR-Tóol* is minimalist and stable, with no movement sensation, rotating cameras, or speedy scenes. Thus, we consider that such characteristics contributed to the low rates of reported symptoms. The motivation and satisfaction questionnaire we created to complement the data showed consistent information with the collected results. Most of the participants considered the *VR-Tóol* task enjoyable, likeable, recommendable, and comfortable. Quantitative and qualitative data showed a general positive VR experience with *VR-Tóol,* thus supporting a part of our first hypothesis, which assumed adequate usability and acceptability parameters of *VR-Tóol*. However, the sense presence assessment reached marginally acceptable parameters and non-acceptable scores in the Involving subscale, thus, we partially accept the first hypothesis.

### *VR-Tóol* performance

The second aim of the study was to explore and describe the performance of a setup task using *VR-Tóol.* Such performance was analyzed in terms of hits, misses, and response times. In our task, a rate of 83.3% of total hits was achieved, which is comparable with the accuracy rate observed in other ER tasks like The Emotion Recognition Task (TER) from CANTAB, in which healthy participants with similar age and educational attainment reached an accuracy rate range between 70% and 80% [58]. Our results are also comparable with the Ekman 60 Faces Test, in which the hit rate has been reported at 80% in healthy individuals [59]. Interestingly, healthy participants at different ages tend to reach an accuracy rate within the 70–80% range in TER at the identification of emotions like happiness, anger, surprise, sadness, and disgust [58,60], whilst in our task, participants reached a hit rate within the 80–97% range for the same emotions when presented at high intensity. Possible explanations for such results could be that *VR-Tóol* includes dynamic facial expressions that resemble the physiognomy of the target sample. In comparison with the percentage of correct responses reported by Del Aguila (90.3%) [22], using an immersive VR ER task, our results yielded a lower percentage of correct responses (83.3%). This discrepancy is likely attributable to the fact that our task involved the manipulation of three levels of intensity. Consequently, if we consider only the percentage of correct responses for emotions with high intensity (90%), our results are found to be highly similar. It is therefore pertinent to consider that the capacity to modulate the intensity of emotional expression might offer advantages in both the assessment and training of ER.

Another interesting finding was that participants were less accurate for the identification of negative emotions like anger, pain, and sadness, reaching a hit rate range of 74–81%. The difficulty in identifying negative emotions when compared to positive emotions has already been reported. Kessels et al. [61] indicated that emotions like happiness tend to reach ceiling effects, while emotions like fear, sadness, or surprise are more difficult to identify, especially if presented in a mild intensity modality. A similar effect was reported by Williams et al. [62], who described that the intensity of the facial expression stimuli affects response consistency among different emotions, whereas happiness can be correctly identified at mild intensity, while fear tends to be accurately identified if presented at high intensity.

An unexpected finding on *VR-Tóol* performance was the high accuracy rates to identify complex emotions like contempt (92% hit rate) and admiration (91.3% hit rate). Both expressions included body gestures which may have played an essential role for ER. It has been proposed that within social interaction processes, non-verbal cues including body gestures, can help to accurately discriminate positive and negative emotions [63]. A recent ER paradigm, the EmBody/EmFace task [64], aims to assess ER through 3D animations using body gestures or facial expressions. Results from this paradigm showed that accuracy rates were similar for emotions like anger or happiness in both facial expressions and body gestures. Thus, we suggest that the concurrent presentation of two non-verbal cues (i.e., facial and body expressions) may facilitate the identification of complex emotions. Taking the performance results together, our second hypothesis is accepted since the obtained *VR-Tóol* scores are similar to those reported using traditional assessment tools.

The response time analysis showed a mean of 10.2 s, which is significantly longer than those reported in other tasks [65]. Such a long response time was due to the task setup in which the registered time included the response of two items per stimulus: the identification of the emotion, followed by the identification of its intensity. Further setups must separate the time registration for each item.

Considering our findings and in accordance to the VR-CHECK framework [10], it is possible to identify several advantages inherent to the *VR-Tóol*. First, it enables the generation of dynamic facial expressions of basic and complex emotions, including body movements in some emotions. Additionally, it allows for the generation of different types of responses (e.g., forced multiple choice, central affect dimensions, and free verbal response), which enhances ecological relevance. Another advantage of *VR-Tóol* is that it permits the generation of emotions with three different levels of intensity, which enables a more comprehensive analysis and greater adaptability of the task. Another crucial feature of *VR-Tóol* is its capacity to generate automated protocols or stimuli in real time, which allows for repetitive application, immediate feedback, and modification of difficulty levels, thus enhancing the feasibility of training. In contrast, the findings demonstrate that *VR-Tóol* exhibits immersive capabilities and technical and user feasibility within acceptable parameters. However, it is important to note that the task presents certain limitations that require further attention. Primarily, the task features a single VR scenario with a female avatar only, and only complex emotions are accompanied by body movements. These limitations will be addressed in future studies and versions.

The development of flexible and dynamic VR-ER tasks implies the potential to have a significant impact in clinical and research contexts, both for the assessment and rehabilitation of ER deficits in multiple disorders. However, certain aspects must be prioritized to ensure optimal adaptability to clinical environments. These include: 1) the incorporation of a more extensive array of dynamic emotions and levels of intensity; 2) the incorporation of corporal movements in the emotional expression; 3) the ability to automatically record responses and response time; 4) the possibility of having different types of response (i.e., multiple-choice, dimensional, free verbal response); and 5) the flexibility of the software to perform automated protocols or generate stimuli in real time. These aspects have the potential to overcome some of the limitations inherent in traditional approaches to ER assessment and treatment.

### ER and empathy

Finally, we aimed to analyze the association between *VR-Tóol* performance and scores on an empathy questionnaire due to the theoretical relationship between both constructs. As mentioned above, empathy seems to be closely related to ER, whereas the latter may be a prerequisite for the former to take place adequately [6]. Following this assumption, we tested if ER and empathy were significantly associated. Results showed that *VR-Tóol* hits were significantly associated with TECA’s total score and PT subscale.

These results are consistent with the assumption that ER is a basic process that enables more complex processes – like empathy – to take place. The cognitive model of empathy proposed by Bird and Viding [6] suggests that general (i.e., Situational understanding system) and specific (i.e., Affective cue classification system) domain mechanisms are involved in the identification of the affective state of others. According to these authors, empathy arises through the integration of the observer’s emotional contagion plus the identification of the emotional state of the other, information provided by the aforementioned mechanisms. Furthermore, empirical evidence has supported the existence of a significant though complex relationship between empathy and ER through diverse methodologies [66–68]. As explained earlier, it was decided to include the PSY domain into the partial correlation analysis due to the strong evidence supporting ER deficits in psychotic disorders, like schizophrenia spectrum disorders. Such deficits have been consistently reported among a wide variety of studies with patients [69,70] and at-risk for psychosis populations [71,72] with moderate to large effect sizes. The correlation analysis results support our third hypothesis, which predicted significant associations between ER and empathy. Nevertheless, these findings are preliminary and must be analyzed with a more robust methodology and objective measures.

## Conclusion

The present study demonstrates that *VR-Tóol* exhibits favorable usability characteristics and an acceptable sense of presence, while also being associated with negligible symptoms of cybersickness. Quantitative and qualitative data showed a generally positive VR experience with *VR-Tóol*. Notably, a substantial accuracy rate was achieved, particularly in identifying emotions such as contempt, admiration, happiness, surprise, and compassion. As expected, emotions with high intensity had a higher percentage of correct responses. In addition, this study shows evidence of the relationship between ER and empathy in healthy subjects. Finally, this work elucidates the potential utility of VR as a tool for facilitating ER assessment.

## Limitations

The present study has some limitations that should be addressed in future studies. We assessed a small sample size, and the experimental design was cross-sectional, so causal inferences among variables cannot be reached. Due to the characteristics of the sample, including its size and distribution, it was decided to omit more robust parametric analyses, such as linear regressions. The reason for this decision is that such analyses would result in an elevated probability of error and a limitation of the generalizability of the results. It also must be noted that the sample was constituted by young adults with high educational attainment, thus preventing us from exploring the variables of interest in a wider heterogeneous sample.

## Future perspectives

The present exploratory study has provided preliminary evidence on the utility of *VR-Tóol* as a potential tool for ER research. Future studies should include wider samples in terms of age and educational attainment. Exhaustive psychometric analyses must be performed to reach robust conclusions about *VR-Tóol*’s reliability and validity for ER assessment. It is imperative that future studies incorporate a variety of responses (dimensional and free verbal responses) using the *VR-Tóol* to enhance the evaluation and ascertain its validity. Finally, subsequent studies may aim to examine the ethical considerations and practical barriers to the implementation of VR training and assessment in clinical and public institutions, with a focus on populations with varying degrees of technological access and cultural backgrounds. This would facilitate the adoption of this technology in practical settings.

## Supporting information

S1 Table Traditional methods for ER assessment. (DOCX)

S2 Table Different paradigms for ER described by Barrett et al. [8]. (DOCX)

S3 File Semi-structured interview protocol. (PDF)

S4 File Motivation and Satisfaction Questionnaire. (PDF)

S5 File SSQ graphic results. (PDF)

S6 File VR-Tóol graphic performance results. (PDF)

S7 File Correlations between VR-Tóol and TECA. (PDF)
