# Supplementary material for: Usability, sense of presence, and performance of a virtual reality emotion recognition task
Source: PLoS One. 2025 Aug 12;20(8):e0330084. doi: 10.1371/journal.pone.0330084 (PMC12342317; doi:10.1371/journal.pone.0330084)
Supplement: S1 Table — (DOCX) [file pone.0330084.s001.docx]

**Supplemental material**

**S1. Traditional methods for ER assessment.**

| **Domain** | **Type of stimuli** | **Examples** |
| --- | --- | --- |
| **ER facial expressions** | Photographs | Picture of Facial Affect (Ekman y Friesen, 1976) |
|  | Microexpression | Ecological Microexpression Recognition Test (Zhang et al., 2017) |
|  | Videos | Facial and Body Expression Recognition (Leyva, 2017) |
| **ER**  **postures** | Photographs | Bodily Expressive Action Stimulus Test- BEAST (de Gelder y Van den Stock, 2011) |
|  | Videos | Facial and Body Expression Recognition (Leyva, 2017) |
| **ER prosody** | Audios | Diagnostic analysis of Nonverbal Accuracy Adult Paralanguage - DANVA2-AP (Nowicki y Duke, 1994) |

ER = Emotion recognition
