# Supplementary material for: Usability, sense of presence, and performance of a virtual reality emotion recognition task
Source: PLoS One. 2025 Aug 12;20(8):e0330084. doi: 10.1371/journal.pone.0330084 (PMC12342317; doi:10.1371/journal.pone.0330084)
Supplement: S2 Table — (DOCX) [file pone.0330084.s002.docx]

**S2 Table. Different paradigms for ER described by Barrett et al. [9].**

| **Paradigms** | **Description** |
| --- | --- |
| **Multiple/forced choice** | Matching pictures of facial configurations and emotion words (with or without short stories). Response options are limited to those provided in the task. |
| **Free classification** | Photos of facial configurations are classified into groups, so that each group represents a perceived category. |
| **Clue matching** | Photos of facial configurations are paired with a recording of posed vocalization. |
| **Similarity tasks** | Judgments between pairs of facial configurations |
| **Perceptual matching** | Indicate whether two photos of facial configurations belong to the same emotion category. |
| **Free response** | Photos of facial configurations are labeled with words provided by the participants (no restrictions on the part of the experimenter). |

ER = Emotion recognition
