# Supplementary material for: Usability, sense of presence, and performance of a virtual reality emotion recognition task
Source: PLoS One. 2025 Aug 12;20(8):e0330084. doi: 10.1371/journal.pone.0330084 (PMC12342317; doi:10.1371/journal.pone.0330084)
Supplement: S3 File — (PDF) [file pone.0330084.s003.pdf]

Fecha: _____/_____/_____

Datos Demográficos

Nombre: ____

Edad: ________ Género: ________ Escolaridad (años): ________

Estado civil: ___________________ Ocupación: ____________________

Lugar de residencia: _______________ Teléfono: ______________________

Antecedentes familiares psiquiátricos y neurológicos

¿Algún familiar cercano (padres o hermanos) padece o ha padecido las siguientes enfermedades?

Esquizofrenia	SI	NO	Quién ______________________________________
Trastorno Bipolar	SI	NO	Quién ______________________________________
Depresión	SI	NO	Quién ______________________________________
Trastorno de ansiedad	SI	NO	Quién ______________________________________
Epilepsia	SI	NO	Quién ______________________________________
Migraña	SI	NO	Quién ______________________________________
Demencia	SI	NO	Quién ______________________________________
Otro	SI	NO	Quién ______________________________________

Antecedentes psiquiátricos y neurológicos

¿Usted ha sido diagnosticado con alguna de las siguientes enfermedades?

Epilepsia	SI	NO	TDAH	SI	NO
Migraña	SI	NO	Dislexia o trastornos de aprendizaje	SI	NO
TCE	SI	NO	Otro	SI	NO
			Cuál ____________________________		

Medicamentos y consumo de drogas

¿Actualmente usted se encuentra bajo algún tratamiento médico tradicional o alternativo?

SI NO ¿Porqué? ____

¿Cuáles medicamentos consume?
____
____
____
____

¿Fuma tabaco? SI NO
¿Qué cantidad y con qué frecuencia?
____
¿Consume alcohol? SI NO

¿Qué cantidad y con qué frecuencia?
____

¿Consume alguna de las siguientes sustancias?

a.	Marihuana	SI	NO	Frecuencia: _______________________
b.	Cocaína/crack	SI	NO	Frecuencia: _______________________
c.	Heroína	SI	NO	Frecuencia: _______________________
d.	Ácidos/LSD	SI	NO	Frecuencia: _______________________
e.	Inhalantes	SI	NO	Frecuencia: _______________________
f.	Hongos/peyote	SI	NO	Frecuencia: _______________________
g.	Anfetaminas	SI	NO	Frecuencia: _______________________
h.	Otros	SI	NO	Frecuencia: _______________________

Uso de Realidad Virtual

¿Alguna vez has utilizado RV inmersiva? Si ( ) No ( )

¿En qué contexto ha sido?
____
____
____

¿Cuántas veces has utilizado RV?
____
____
____

¿Con que frecuencia has utilizado RV?
____
____
____

En caso de haber utilizado RV anteriormente, ¿Te ha gustado la experiencia?
____
____
____

En caso de no haber utilizado RV anteriormente, ¿Te gustaría ser usuario de RV?
____
____
____

¿Después de usar RV has tenido algún malestar? (mareo, vértigo, dolor de cabeza, nausea, etc.)
____
____
____
