# Supplementary material for: Usability, sense of presence, and performance of a virtual reality emotion recognition task
Source: PLoS One. 2025 Aug 12;20(8):e0330084. doi: 10.1371/journal.pone.0330084 (PMC12342317; doi:10.1371/journal.pone.0330084)
Supplement: S4 File — (PDF) [file pone.0330084.s004.pdf]

Cuestionario de motivación y satisfacción

Instrucciones: Para cada uno de los siguientes enunciados, selecciona la opción que mejor describa tu reacción ante el programa de realidad virtual que probaste el día de hoy

1. ¿Qué tanto te gustó/agradó el ambiente virtual?	Nada	Mucho
2. ¿Qué tanto te gustó/agradó participar en la tarea con RV?	Nada	Mucho
3. ¿Qué tanto te gustaría volver a participar en esta tarea con RV?	Nada	Mucho
4. Las expresiones faciales del avatar me parecieron reales	Totalmente en desacuerdo	Totalmente de acuerdo
5. Estoy dispuesto a volver a participar en esta tarea en un futuro	Totalmente en desacuerdo	Totalmente de acuerdo
6. Las expresiones emocionales del avatar me parecieron falsas	Totalmente en desacuerdo	Totalmente de acuerdo
7. Las opciones de respuesta me parecieron adecuadas	Totalmente en desacuerdo	Totalmente de acuerdo
8. Las expresiones emocionales del avatar me parecieron naturales	Totalmente en desacuerdo	Totalmente de acuerdo
9. Presenté algún malestar después de realizar la tarea	Totalmente en desacuerdo	Totalmente de acuerdo
10. Le recomendaría a mis familiares y amigos participar en esta tarea	Totalmente en desacuerdo	Totalmente de acuerdo
11. El formato de las opciones de respuesta me pareció confuso o poco amigable	Totalmente en desacuerdo	Totalmente de acuerdo

12. El ambiente virtual me pareció:

13. La tarea/programa me pareció:

14. ¿Qué fue lo que más te gustó de la tarea?

15. ¿Qué fue lo que menos te gustó de la tarea?

16. ¿Qué le cambiarías a la tarea?

17. Comentarios
