# Supplementary material for: Usability, sense of presence, and performance of a virtual reality emotion recognition task
Source: PLoS One. 2025 Aug 12;20(8):e0330084. doi: 10.1371/journal.pone.0330084 (PMC12342317; doi:10.1371/journal.pone.0330084)
Supplement: S5 File — (PDF) [file pone.0330084.s005.pdf]

Supplemental 5 – Results graphics

SSQ

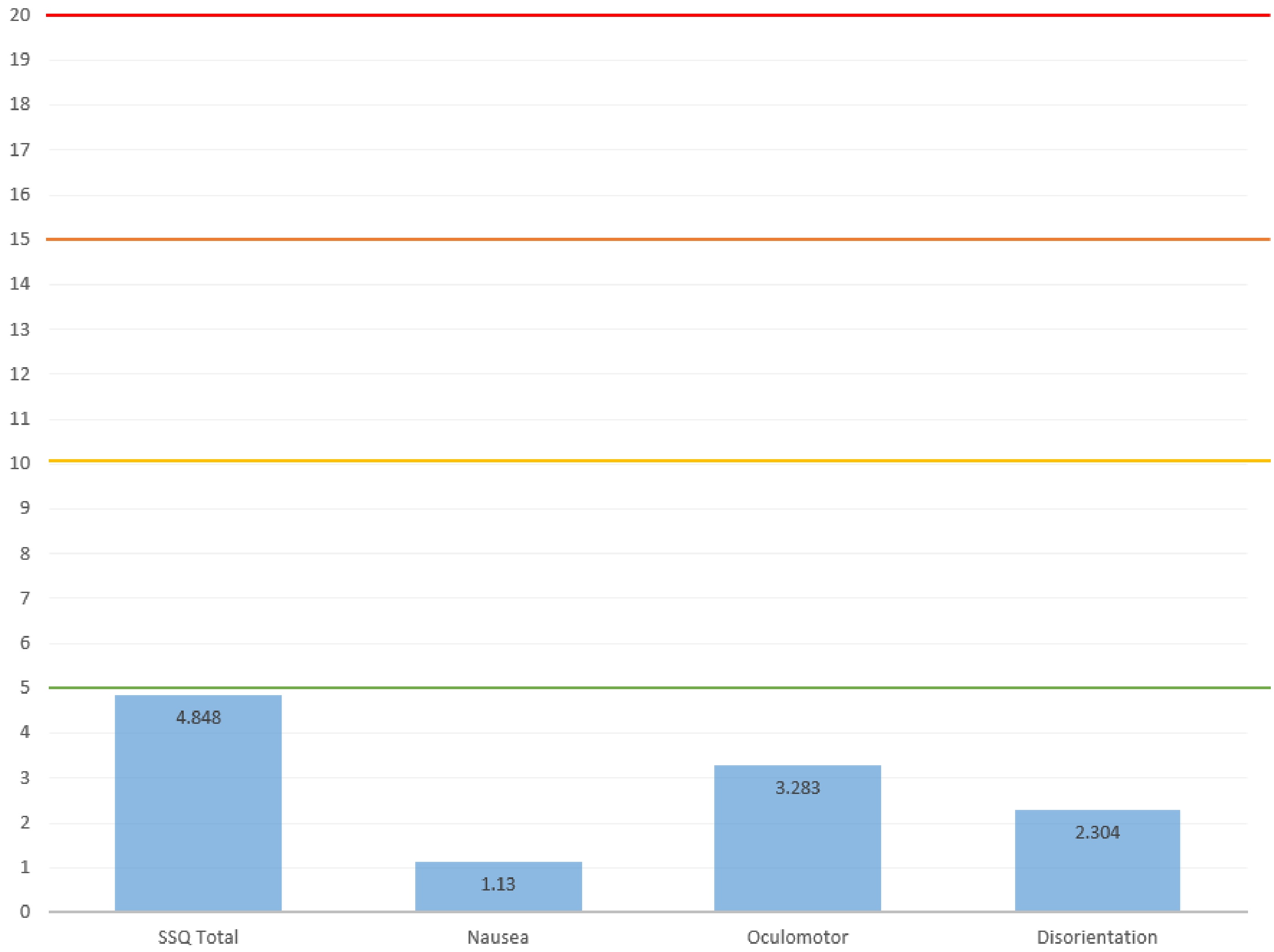

Brown et al. [46] reported the following cut-off points; 0 = No symptoms; ≤ 5 = Negligible symptoms (Green line); 5-10 = Minimal symptoms (Yellow line); 10-15 = Significant symptoms (orange line); 15-20 = Symptoms are a concern (Red line); ≥20 = A bad intervention. The parameters were utilized for the interpretation of the resulting data.

Reference

Brown P, Spronck P, Powell W. The simulator sickness questionnaire, and the erroneous zero baseline assumption. Front Virtual Real. 2022;3.
