# Supplementary figures and images for: Usability, sense of presence, and performance of a virtual reality emotion recognition task
Source: PLoS One. 2025 Aug 12;20(8):e0330084. doi: 10.1371/journal.pone.0330084 (PMC12342317; doi:10.1371/journal.pone.0330084)

## Supplemental 6 - VR-Tóol descriptive results graphics

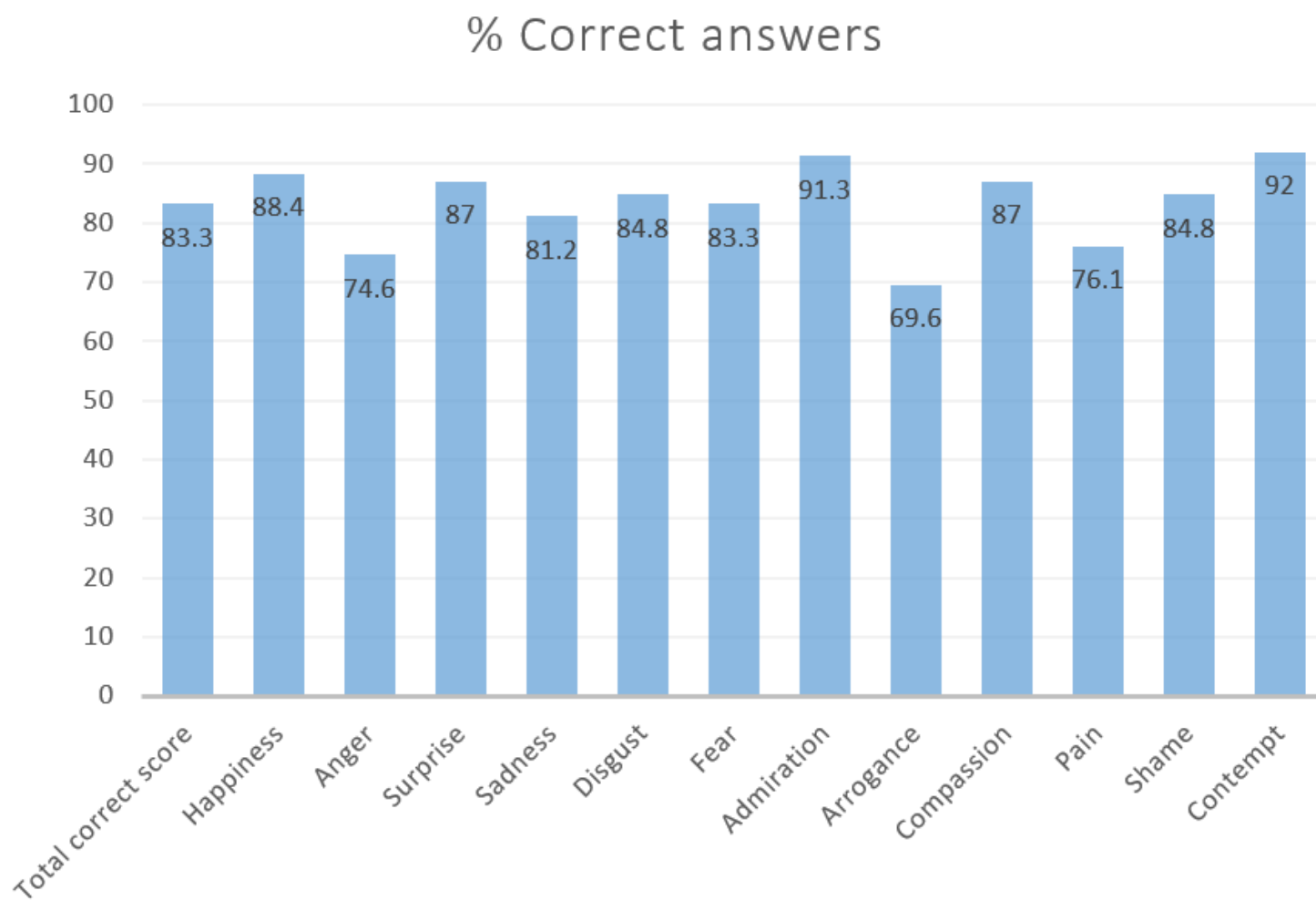

## VR-Tóol descriptive results graphics

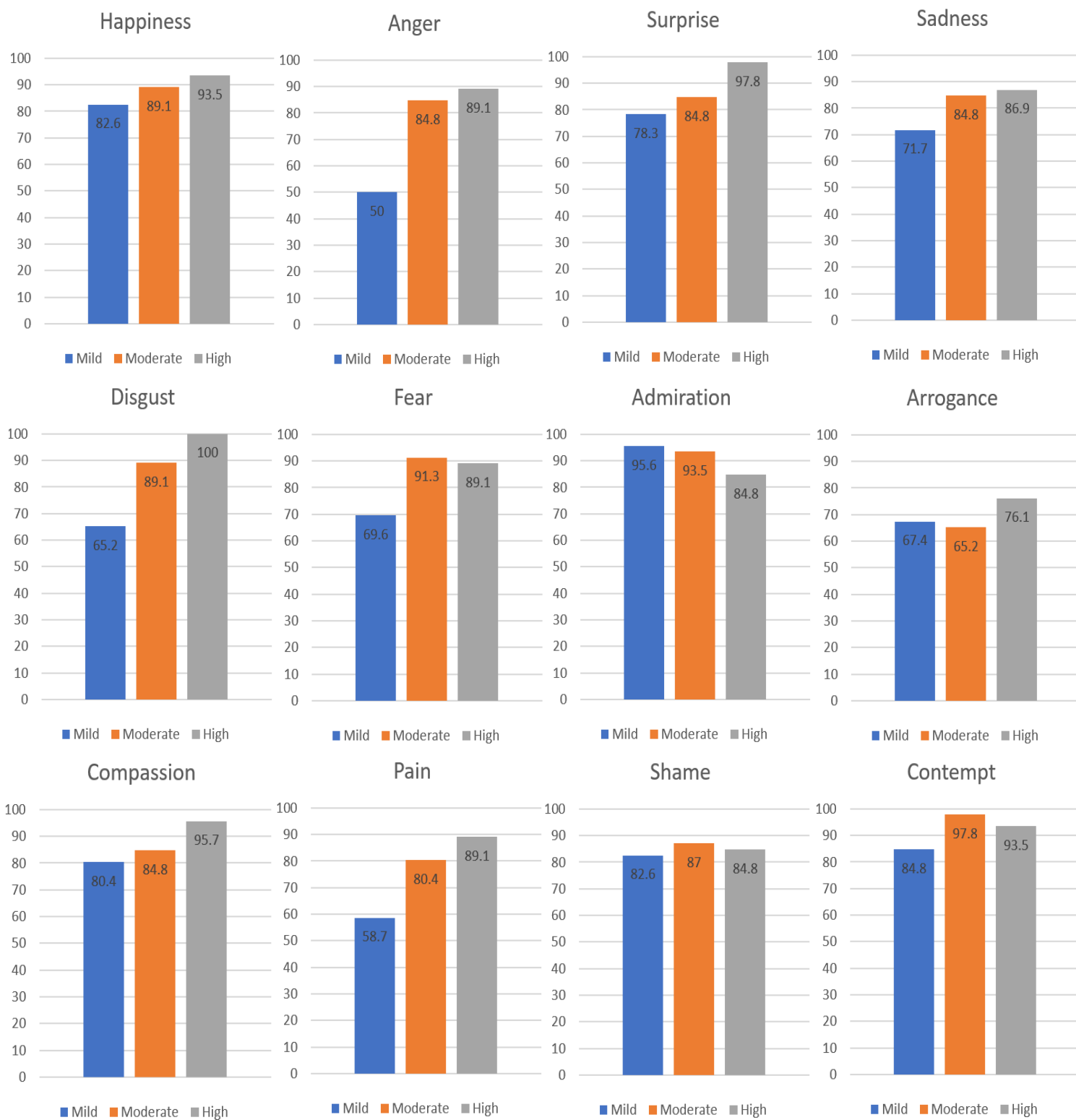

Supplement: S6 File — (PDF) [file pone.0330084.s006.pdf]

**VR-Tóol correct – TECA total**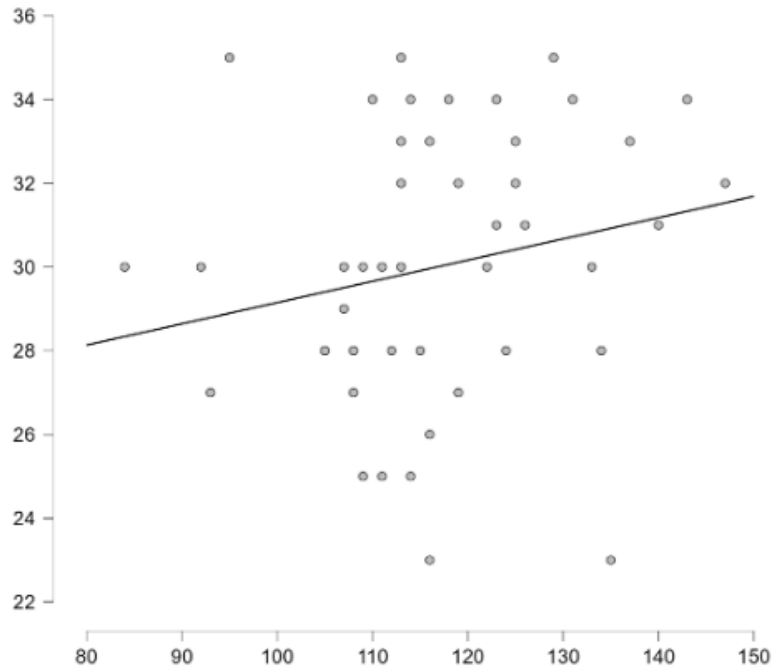

### VR-Tóol intensity – TECA PT

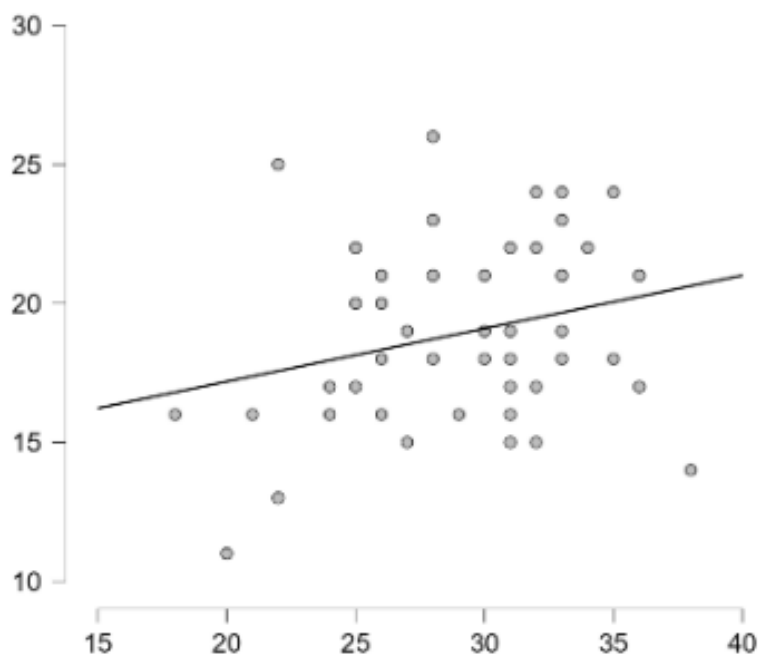

### VR-Tóol time – Age

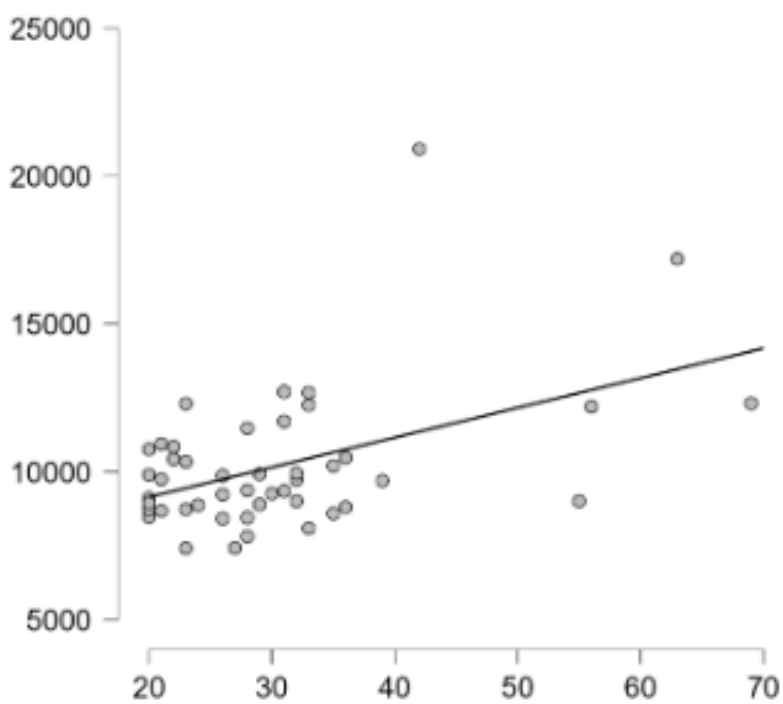

Supplement: S7 File — (PDF) [file pone.0330084.s007.pdf]
